# Exploring early steps in biofilm formation: set-up of an experimental system for molecular studies

**DOI:** 10.1186/s12866-014-0253-z

**Published:** 2014-09-30

**Authors:** Marc Crouzet, Caroline Le Senechal, Volker S Brözel, Patricia Costaglioli, Christophe Barthe, Marc Bonneu, Bertrand Garbay, Sebastien Vilain

**Affiliations:** University Bordeaux, BPRVS, EA 4135, F-33000 Bordeaux, France; Bordeaux INP, BPRVS, EA 4135, F-33000 Bordeaux, France; Department of Biology & Microbiology, South Dakota State University, Brookings, SD 57007 USA; Department of Microbiology and Plant Pathology, University of Pretoria, Pretoria, 0083 South Africa; Université de Bordeaux, Centre Génomique Fonctionnelle de Bordeaux, Plateforme Protéome, Bordeaux, F-33000 France; ENSTBB, 146 rue Léo Saignat, case 87, 33076 Bordeaux cedex, France

**Keywords:** *Pseudomonas aeruginosa*, Glass wool, Adsorption mode, Adhesion, Biofilm

## Abstract

**Background:**

Bacterial biofilms are predominant in natural ecosystems and constitute a public health threat because of their outstanding resistance to antibacterial treatments and especially to antibiotics. To date, several systems have been developed to grow bacterial biofilms in order to study their phenotypes and the physiology of sessile cells. Although relevant, such systems permit analysis of various aspects of the biofilm state but often after several hours of bacterial growth.

**Results:**

Here we describe a simple and easy-to-use system for growing *P. aeruginosa* biofilm based on the medium adsorption onto glass wool fibers. This approach which promotes bacterial contact onto the support, makes it possible to obtain in a few minutes a large population of sessile bacteria. Using this growth system, we demonstrated the feasibility of exploring the early stages of biofilm formation by separating by electrophoresis proteins extracted directly from immobilized cells. Moreover, the involvement of protein synthesis in *P. aeruginosa* attachment is demonstrated.

**Conclusions:**

Our system provides sufficient sessile biomass to perform biochemical and proteomic analyses from the early incubation period, thus paving the way for the molecular analysis of the early stages of colonization that were inaccessible to date.

**Electronic supplementary material:**

The online version of this article (doi:10.1186/s12866-014-0253-z) contains supplementary material, which is available to authorized users.

## Background

Bacteria form complex multicellular structures called biofilms [1]. Biofilm formation is commonly considered to occur in four main stages: (1) bacterial attachment to a surface, (2) microcolony formation, (3) biofilm maturation and (4) detachment (also termed dispersal) of bacteria which may then colonize new areas [2]. Bacteria within the biofilm, termed sessile bacteria, exist in a stationary or dormant growth phase [3] and exhibit phenotypes that are distinct from planktonic bacteria [4]. In biofilms, bacteria display an exceptional resistance to environmental stresses, especially antibiotics [5]. This makes biofilms a major public health problem as 60-80% of human microbial infections are caused by bacteria growing as a biofilm [6,7]. The identification of biochemical pathways and biological factors critical to biofilm formation is important to prevent biofilm formation. Even with our general understanding of the basic structure and development of bacterial biofilms, knowledge of the underlying processes responsible for inducing the transition from planktonic to sessile cells is still unclear. This transition is thought to be a complex and highly regulated process resulting in a phenotypic change [8].

To identify the biological elements of sessile bacteria involved in biofilm physiology and especially in antibiotic resistance, studies were developed in multiple ways using several *in vitro* systems and surfaces [9]. The simplest biofilm system is to set a liquid in a recipient and let the bacteria colonize the solid surface, as described by Zobell [10]. Nowadays, multi-well plates are commonly used in this way to grow and quantify biofilms [11]. Another technique is to add a substratum to a planktonic batch culture (named in this study “the immersion mode”). These systems are cost-effective and simple to implement, but the biofilms formed become increasingly heterogeneous over time. For instance, after 24 h of incubation, the biofilm is composed not only of elderly sessile cells but also of cells recently attached to the surface. In addition, sessile cells are potentially under the influence of surrounding planktonic cells [12], which may impact the results of the study. The latter issue could be solved by using a biofilm system in “flow-through” mode, meaning that the substratum to which the bacteria adhere is immersed in a continuous flow of culture medium [13]. Flow-through systems need specialized equipment and often do not produce the large biomass essential for biochemical studies, except by multiplying the assays or increasing the adhesion surface, which may increase the heterogeneity of the population.

In addition to the variety of approaches, several surfaces with different physicochemical properties such as silicone, clay, metal, hydroxyapatite, polystyrene, polycarbonate and glass have been used to grow biofilms. Borosilicate glass has been validated by the American Society for Testing and Materials (ASTM) Committee (Surface Method E2871 -12) to study the effectiveness of disinfectants on biofilms (http://www.biofilm.montana.edu/content/astm-approves-method). Glass beads or glass wool fibers have been used in flow-through systems [14] or in immersion mode [15]. Glass wool (GW) affords a large surface-to-volume ratio, so a small piece of GW allows the colonization of a large surface area [16], thereby obtaining a large biomass essential for performing biochemical and proteomic analyses. So far, our laboratory has used GW in the immersion mode in a large volume of culture medium [17]. To facilitate biofilm formation and increase the sessile biomass, we investigated the use of GW in adsorption mode rather than in immersion mode. This approach utilizes the high retention capacity of GW, just like a sponge adsorbs a liquid. The rationale was to grow biofilms on the largest surface area with a minimal volume of culture medium adsorbed on GW. As bacteria were in close proximity to GW fibers, the probability for bacteria to encounter the surface was increased, so adhesion to the substratum should be promoted over time. In addition, no cells adhered to the surface of the vessel as biofilms were obtained in a system without contact to container walls. Thus, by using the adsorption mode, we expected to obtain a larger and more homogeneous population of sessile cells.

This paper presents the attachment and growth of *Pseudomonas aeruginosa* PAO1 on GW in the adsorption mode. The liquid adsorbed on GW formed a regular thin sheath around the fibers in which PAO1 grew like a planktonic culture. We showed that the colonization of the GW surface was very fast and depended in part on protein synthesis. The colonization profile was similar in complex and synthetic media. However, it was influenced by the bacterial concentration of the inoculum. Our system provides sufficient sessile biomass to perform proteomic analyses from the early incubation period, thus paving the way for the molecular analysis of the early stages of colonization that were inaccessible to date.

## Methods

### Bacterial strain, growth conditions and biofilm formation

*Pseudomonas aeruginosa* PAO1 (CIP 104116) was provided by the Institut Pasteur (CRBIP, Paris, France). Strain PAO1 was grown either in lysogeny broth (LB: tryptone 10 g/L; yeast extract 5 g/L; NaCl 5 g/L; pH 7.2) or in a synthetic medium (SM: 60 mM K_2_HPO_4_; 30 mM KH_2_PO_4_; 7.5 mM (NH_4_)_2_SO_4_; 1 mM MgSO_4_,7H_2_O; 17 mM glucose; 10 μM FeSO_4_,7H_2_O; pH 7.2) previously described by Aspedon *et al.* [18].

Planktonic and biofilm cultures were performed at 37°C under agitation (150 rpm). Overnight pre-cultures were obtained by inoculating 20 mL of medium with one bacterial colony. Afterwards, bacterial suspensions were prepared by diluting the pre-culture in fresh identical medium at 1/10, 1/100 or 1/1000 corresponding to ≈ 10^9^, ≈10^8^ and ≈ 10^7^ CFU/mL, respectively. Planktonic cultures consisted of incubating 20 mL of bacterial suspension in a 100 mL Erlenmeyer flask. Biofilms were grown on GW fibers used either in immersion or adsorption modes. In immersion mode, a 1 g piece of GW was placed in 100 or 500 mL of medium with bacteria. In adsorption mode, 5 mL of bacterial suspension were adsorbed on 1 g GW.

### Glass wool characteristics

Glass wool material was provided by the Sodipro Company (Echirolles, France, ref. number SCI03950). Calibrated pieces of GW (1 g) in distilled water (50 mL) were sterilized by autoclaving (121°C, 20 min). Water was removed by vacuum aspiration and the pieces were dried for 48 h at 80°C before use. The density of the GW fibers was determined experimentally. The mass of several randomly cut GW pieces was determined and their volume was estimated by measuring the volume increase after immersion in a known volume of water. The diameter of the fibers was directly determined by microscopy. In adsorption mode, the maximum volume that could be immobilized on a 1 g piece of GW was determined experimentally by adding mL per mL of liquid (water, LB and SM media) until liquid leaked. Then the value was refined by adding 0.1 mL per 0.1 mL until a drop appeared. Finally, the surface covered by the adsorbed volume was defined by using an aqueous solution of methylene blue 0.02% (w/v). The ratios tested were 10, 7.5, 5, 2.5 and 1 mL / g GW. After adsorption, GW pieces were placed in 100 mL Erlenmeyer flasks and were incubated at 37°C for 6 h under agitation (150 rpm). Then the stained parts were cut off and the colored and uncolored parts were dried for 48 h at 80°C. The percentage of covered surface was calculated by measuring the mass of colored and non-colored parts. The percentage of covered surface was also determined just after liquid adsorption onto GW by performing the same experiment without the 6 h incubation period.

### Construction of PAO1 expressing eGFP

A PAO1 strain expressing eGFP was constructed for this study. The eGFP coding sequence was amplified from the plasmid pEGFP (Clontech, CA, USA) using the primers 5′-ATGGTGAGCAAGGGCGAGGAGCTGTTCACC-3′ and 5′-TT*CTGCAG*AGTCGCGGCCGCTTTACTTGTAC-3′ (containing a *Pst*I restriction site at 3′ end). The eGFP coding sequence was set under the control of the promoter region of the PA4249 gene. This gene has been shown to be constitutively expressed in planktonic and sessile PAO1 cells [17]. The PA4249 promoter was amplified from PAO1 genomic DNA using the primers 5′- AA*GGATCC*CAAGTTCGGCCTGAGCCGTAACAA-3′ (containing a *Bam*HI restriction site at 5′ end) and 5′- TTGCTCACCATGGGCTTAACGCTCCTGATAC-3′. All primers were provided by Eurogentec. PCR cycles were done as follows: denaturation 95°C, 30 s; annealing 63°C, 45 s; elongation 72°C, 1 min performed with a Phusion Taq polymerase (New England Biolabs, MA, USA). The two amplicons were fused and the resulting DNA fragment carrying the construction - PA4249 promoter - eGFP CDS (named pPA4249-eGFP below) - was amplified and purified from agarose gel. The fragment was cloned into pUCP20 (kindly provided by Dr. Schweizer) by *Pst*I - *Bam*HI double digestion. pUCP20 is a high-copy plasmid replicating in *E. coli* and *P. aeruginosa* [19]. PAO1 was transformed by the plasmid pUCP20-[pPA4249-eGFP] according to a protocol previously described [20] and transformants were selected on LB agar with carbenicillin 200 μg/mL. GFP fluorescence arising from transformants (515 nm) was checked with a Versafluor fluorometer (Biorad). The recombinant DNA was verified by DNA sequencing. The plasmid pUCP20-[pPA4249-eGFP] allowed the constitutive eGFP expression in strain PAO1 enabling bacteria to be self-labeling.

### Flow-through washing process and bacterial quantification

One g pieces of GW used in immersion or adsorption modes were placed in a 50 mL syringe such that the fibers were parallel to the vertical axis of the syringe. Washing was performed with 100 mL PBS (NaCl 8 g/L, KCl 0.2 g/L, Na_2_HPO_4_ 2H_2_O 1.44 g/L, KH_2_PO_4_ 0.24 g/L) running down through GW by gravity. This step was completed in less than 40 seconds (volumetric flow rate = 2.6 ± 0.1 mL.s^−1^). The planktonic and loosely attached bacteria were recovered in the flow-through. The GW piece was removed from the syringe and placed in 100 mL PBS. Sessile cells were harvested from GW by vortexing vigorously for 30 seconds. GW was then squeezed against the wall of the flask. To achieve maximum recovery of bacteria, this latter step was repeated three times in the same PBS bath [17]. Ultimately the squeezed GW was discarded. The planktonic and sessile bacterial biomasses contained in PBS solutions were quantified by colony forming unit (CFU) counting. The number of CFU was determined by plating 0.1 mL aliquots of serial dilutions twice onto LB agar and incubating for 24 h at 37°C. All time points were performed in biological triplicate.

### Spinning-disk microscopy

Microscopy experiments were performed on 1.5 mg pieces of GW loaded with 3.75 or 7.50 μL of LB (*i.e.* 2.5 or 5 mL/g GW) with or without bacteria (10^7^ CFU/mL). Cell attachment and biofilm development, as well as the diameter of GW fibers, were determined by spinning-disk microscopy. The spinning-disk experiments were done on an inverted Leica DMI 6000 microscope (Leica Microsystems, Wetzlar, Germany) equipped with a confocal head Yokogawa CSU-X1 (Yokogawa Electric Corporation, Tokyo, Japan) and a resolutive HQ2 camera (Photometrics, Tucson, USA). The diode laser used was at 491 nm. The objective used was a HCX PL Fluotar 40X oil 1.25 NA or HCX PL APO CS 63X oil 1.32 NA. The z stacks were performed with a piezo P721.LLQ (Physik Instrumente (PI), Karlsruhe, Germany). The mosaics were done with a motorized stage Scan IM (Märzhäuser, Wetzlar, Germany). The 37°C atmosphere was created with an incubator box and an air heating system (Life Imaging Services, Basel, Switzerland). This system was controlled by MetaMorph software (Molecular Devices, Sunnyvale, USA). The microscopic experiments were performed three times and more than twenty five frames were observed for each experiment.

### Tetracycline effect on initial colonization of glass wool by P. aeruginosa

The study of tetracycline effect on *P. aeruginosa* adhesion was inspired from data reported by O’Toole and Kolter [21]. Stationary and exponential calibrated bacterial suspensions (10^8^ CFU/mL) were prepared from an overnight culture. Briefly, stationary cells were obtained by diluting pre-culture overnight in LB (1:100). Exponential cells were obtained by inoculating fresh LB with pre-culture and incubated up to OD_546nm_ = 0.3. These bacterial suspensions were treated or not for 1 h at the bacteriostatic concentration of tetracycline (*i.e.* 10 μg/mL and 150 μg/mL for exponential and stationary cultures, respectively). After antibiotic treatment, 5 mL were adsorbed on 1 g of GW incubated at 37°C for 20 min and sessile bacteria were quantified as mentioned above. The same experiment was performed using the microplate biofilm formation assay as previously described [11]. Briefly, the diluted cultures were incubated in wells of microplates and biofilms formed after 20 min were quantified by staining with crystal violet.

### Protein extraction and electrophoresis

The objective of this experiment was as follows: (1) to extract the protein content of relatively few planktonic or sessile bacteria (10^8^ and 10^9^ CFU); (2) to maintain the integrity of the proteome by directly lysing bacteria *in situ*; (3) to demonstrate the feasibility of the approach using GW. To obtain 10^8^ sessile cells, 5 mL of LB at 10^7^ CFU/mL were adsorbed onto 1 g of GW. After 3 h of incubation at 37°C, GW was washed as mentioned above (see “Flow-through washing process and bacterial quantification”). PBS was immediately removed by pipetting, leaving ≈ 3 mL adsorbed on GW. The same number of planktonic cells was obtained from 170 μL of a 3 h-old LB planktonic culture (see “Bacterial strain, growth conditions and biofilm formation”). Similarly, 10^9^ sessile cells were obtained by adsorbing 5 mL of LB at 10^9^ CFU/mL onto 1 g of GW. After 1 h of incubation at 37°C, GW was washed and PBS was removed, leaving ≈ 3 mL on GW. The same number of planktonic cells was obtained from 500 μL of a 1 h LB planktonic culture.

Cell lysis was performed by adding one volume of lysis buffer 2X (7 M urea, 2 M thiourea, 65 mM CHAPS, 20 mM DTT, 1 M NaCl) to one volume of sample. The mix was frozen (-80°C, 30 min) and thawed (35°C, 20 min). The proteins were concentrated by 15% TCA precipitation followed by two successive acetone washings. Proteins were suspended in 50 μL of the following solution: 7 M urea, 70 mM SDS, 20 mM DTT. Nine μL of protein extract were mixed with 3 μL of Laemmli buffer 4X and then 10 μL were loaded on an SDS-PAGE (12%). After electrophoresis, proteins were visualized by colloidal Coomassie blue staining. The gels were scanned with a GS-800 densitometer (BioRad).

## Results

### Culture medium formed a sheath surrounding the glass wool fiber in adsorption mode

**Figure 1:**
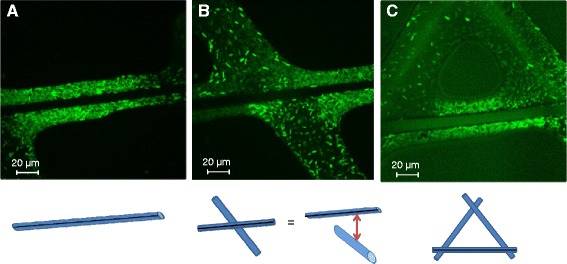
**Microscopy observations of the inoculated medium adsorbed on glass wool.** The calculated thickness [see Additional file 3] was confirmed by confocal microscopic observation of several pieces of GW loaded with a ratio of 5 mL/g GW **(A, C)** or 2.5 mL/g GW **(B)** of LB containing PAO1. In any case, we observed a minimal thickness of ≈ 15 μm even when several fibers were intertwined **(C)**. The black line on schematic representation indicates the fibers on the focal plane.

In this study, biofilms were cultivated on rope-shaped borosilicate glass wool (GW) cut in cylinders (Ø = 4 cm; 2.5 cm high) and weighing 1 g [see Additional file 1A]. GW density was evaluated experimentally to be 1154.7 ± 57.6 kg/m^3^ (n = 4) and fiber diameter was determined to be 10 μm by microscopic observation (n = 10) [see Additional file 1B]. In other terms, 1 g of GW could be seen schematically as a 11 × 10^3^ m-long cylinder with a diameter of 10 μm offering a large 3464 cm^2^ colonization surface in a limited space [see Additional file 1C]. As the “adsorption mode” was used in this study, we first determined the percentage of covered surface as a function of the volume adsorbed on GW by using methylene blue solution [see Additional file 2]. A 1 g piece of GW adsorbed a maximum of 10 mL, the addition of more liquid leading to leakage from the GW (n = 5). With 10 and 7.5 mL, the whole GW surface was covered. Smaller volumes partially covered the surface. A volume of 5, 2.5 and 1 mL covered 61.0 ± 1.4%, 30.0 ± 2.0% and 13.7 ± 0.8% of the 3464 cm^2^, respectively (n = 6) [see Additional file 2]. These experimental data used with the hollow cylinder model [see Additional file 3A] allowed us to determine that the liquid surrounding the fibers had a minimal thickness of 13 μm [see Additional file 3B]. We verified this calculation experimentally by microscopy performed with small pieces of GW at the ratio of 5 mL (Figure 1A,C) or 2.5 mL (Figure 1B)/g of GW by using LB containing *P. aeruginosa* (10^7^ CFU/mL) or not. In every case, the medium was homogeneously distributed along the fiber, including sites where several fibers were intertwined (Figure 1C). In agreement with the calculated value, the average thickness of the sheath formed around the fibers was ≈ 15 μm. Thus, the adsorption mode allowed a large surface of GW to be surrounded by a thin layer of liquid.

### Discrimination of sessile bacteria from planktonic bacteria

In immersion systems, colonized surfaces like GW are removed from the batch and washed to separate the biofilm biomass from the planktonic bacteria. For our adsorption system, we developed a new approach to separate strongly attached bacteria from planktonic/ weakly attached bacteria without excessive handling of the material. The removal of planktonic and weakly attached bacteria was performed through washing the GW. This was achieved by flowing 20 volumes of PBS solution through the GW for one adsorbed volume, *i.e.* 100 mL of PBS for 5 mL adsorbed on GW [see Additional file 4]. For that purpose, GW was placed in a 50 mL syringe and flushed with PBS by gravity at a volumetric flow rate of 2.6 ± 0.1 mL.s^−1^ (n = 9). Finally, the number of bacteria in the flow through and retained on the washed GW fibers was determined as described in Methods section. The efficiency of the process was tested with a 1 g piece of GW inoculated with 5 mL of complex (LB) or synthetic (SM) medium at three different bacterial concentrations (10^7^, 10^8^ or 10^9^ CFU/mL). When the washing step immediately follows the adsorption step, the PBS “flow-through” contained more than 98% of CFU of the inocula (data not shown), thereby proving the effectiveness of this method to remove all unattached bacteria. This result was validated by microscopic observations since no adhered bacteria were observed on GW (data not shown). When the inoculated GW pieces were incubated for 24 h, 5.3 ± 0.7 10^6^ CFU/cm^2^ were still present on the GW after the PBS washing step. Microscopic observations of these GW pieces showed that the retained bacteria were not motile and, either in contact with the surface or with bacteria which were immobilized on the GW fiber. In our study, these bacteria that were not removed after PBS washing were then considered as adhered/sessile cells. So, the flow-through process was appropriate to estimate the sessile bacterial population and thus to study the GW colonization by *P. aeruginosa*.

### P. aeruginosa grew in adsorption mode as in a standard planktonic culture…but survived longer in the former

Surface colonization and biofilm development respond to various signals such as the nutritional conditions of the environment [11]. As the adsorption mode may influence bacterial growth and therefore biofilm formation, we examined *P. aeruginosa* growth in complex (LB) or synthetic (SM) medium adsorbed on glass wool by determining the number of CFU, without distinguishing unattached cells from sessile cells. These results were compared with planktonic cultures (PC) at identical initial bacterial concentrations in the same media. As this study was performed with 5 mL of medium initially at 10^7^ or 10^9^ CFU/mL adsorbed on a 1 g piece of GW, we compared total CFU from cultures on GW with CFU number obtained in 5 mL of PC.

**Figure 2:**
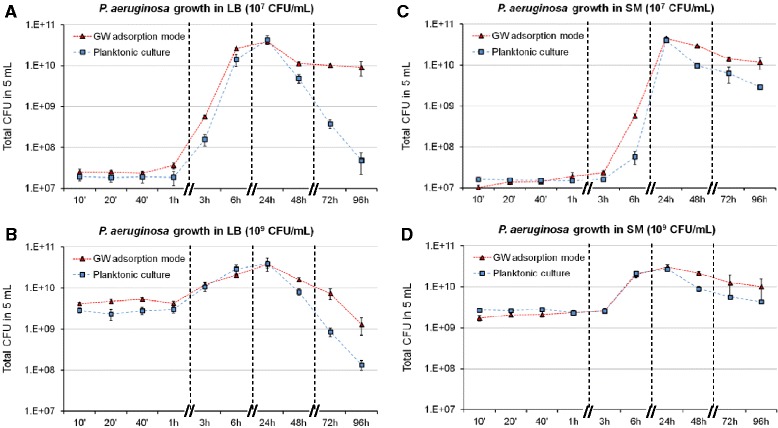
**Total CFU over time in adsorption mode (GW) versus planktonic culture.** The LB **(A,B)** or SM **(C,D)** cultures were inoculated at 10^7^ **(A,C)** or 10^9^ **(B,D)** CFU/mL. One g of GW was inoculated with 5 mL of medium. After incubation, total CFU (planktonic and sessile cells) on GW were compared to CFU contained in 5 mL of planktonic culture. Each point is the mean ± SD of biological triplicates. A non-linear time scale was chosen and dotted lines were drawn between experimental points for the sake of clarity.

In LB, there was a lag phase lasting around 1 h in both PC and GW cultures, irrespective of the initial bacterial concentration (Figure 2A,B). In SM, the lag phase lasted around 3 h (Figure 2C,D). Then, in both media, the biomass increased up to 24 h, the time point at which viable biomasses were all equivalent (Figure 2). This indicates that neither the growth mode nor the initial bacterial concentration had any influence on the 24 h-old biomass. Nevertheless, during the growth phase in LB or SM inoculated at 10^7^ CFU/mL, a slight difference was observed between CFU in PC and GW cultures (Figure 2A,C). For instance, the CFU ratio between GW and PC at 3 h in LB was 3.6 and the average doubling time between 1 h and 3 h of incubation was 39 min in PC and 30 min in GW culture. More strikingly, the CFU ratio between GW and PC at 6 h in SM was 10.0 and the average doubling time between 3 h and 6 h of incubation was 65 min in PC and 26 min in GW culture. These differences were not observed in LB or SM inoculated at 10^9^ CFU/mL (Figure 2B,D).

After 24 h, the viability decreased in both PC and GW cultures but differently according to the medium. In LB-PC, the biomass was reduced by a factor ≈ 3 log at 96 h compared to the biomass at 24 h, irrespective of the initial bacterial concentration (Figure 2A,B). In GW culture, the viability depended on the initial bacterial concentration. At 10^7^ CFU/mL, the reduction was limited to the 24 h-48 h period, and then the number of viable bacteria remained stable (≈10^10^ CFU) (Figure 2A). At 10^9^ CFU/mL, the biomass continually decreased up to 96 h (Figure 2B). In any case, the 72 h- and 96 h-old biomasses in GW cultures were greater by a factor > 1 log than the biomasses in PC, indicating that GW culture enhanced the long-term survival of *P. aeruginosa*. In SM-PC from 48 h to 96 h, whatever the initial bacterial concentration, we systematically observed the clumping of bacteria resulting in a huge free-floating aggregate in the medium. This prevented us from comparing the PC and GW biomasses. Even if a strict comparison was not possible, a long-term survival was observed when *P. aeruginosa* was grown in SM.

Thus growing bacteria in a limited volume of LB or SM medium adsorbed on GW had no negative effects on *P. aeruginosa* growth compared to a planktonic culture grown in the same conditions. The biomasses obtained after 24 h of incubation were similar in all conditions (PC *vs* GW, LB *vs* SM). Moreover, consistently with the properties of sessile cells in biofilms classically described in the literature [1], the survival of bacteria on GW was higher after several days of culture in comparison to planktonic cells.

### Colonization of GW fibers by P. aeruginosa was very quick and dependent on the bacterial concentration

**Figure 3:**
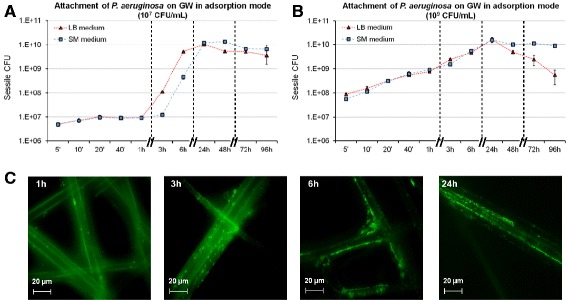
**Sessile CFU over time in adsorption mode (GW).** The LB or SM cultures were inoculated at 10^7^ **(A)** or 10^9^ **(B)** CFU/mL. One g of GW was inoculated with 5 mL and sessile cells were quantified over time (see Methods section). Each point is the mean ± SD of biological triplicates. A non-linear time scale was chosen and dotted lines were drawn between experimental points for the sake of clarity. **(C)** Attachment and biofilm development on GW fibers in LB inoculated at 10^7^ CFU/mL. Before observation, GW was washed as mentioned in methods section. Pictures are Z-stack of 20 μm depth images obtained by confocal microscopy. Attached bacteria were isolated cells at 1 h and 3 h, whereas they formed microcolonies as of 6 h incubation.

After studying the total biomass grown on GW over time, we focused on the sessile part of this biomass obtained in the culture conditions presented above. Briefly, 5 mL of LB or SM medium at 10^7^ or 10^9^ CFU/mL were adsorbed on 1 g of GW. After incubation at 37°C, GW was washed by the flow-through process and sessile bacteria were harvested and quantified as indicated in Methods section. At 10^7^ CFU/mL in LB or SM, the viable sessile bacteria were already 4.8 ± 0.6 10^6^ CFU (2.3 ± 0.3 10^3^ CFU/cm^2^) after only 5 min of incubation. The population doubled between 5 and 20 min up to 1.0 ± 0.2 10^7^ CFU (4.8 ± 0.7 10^3^ CFU/cm^2^) and then remained relatively constant up to 1 h of incubation in LB or 3 h in SM (Figure 3A). After this lag-like phase, the number of viable sessile bacteria continuously increased, reaching a maximum at 24 h, where 1 g of GW carried 1.1 ± 0.2 10^10^ CFU (5.3 ± 0.7 10^6^ CFU/cm^2^), irrespective of the culture medium. Then up to 96 h, the sessile biomass remained relatively stable in SM but slightly decreased in LB (Figure 3A). In LB or SM at 10^9^ CFU/mL, the viable sessile bacteria at 5 min were 7.6 ± 1.8 10^7^ CFU (3.6 ± 0.9 10^4^ CFU/cm^2^) (Figure 3B). Then the sessile biomass continuously increased for 24 h without any difference between LB and SM. After 1 h, the number of attached bacteria increased by one log and at 24 h, the viable sessile biomass was 1.6 ± 0.4 10^10^ CFU (7.4 ± 1.8 10^6^ CFU/cm^2^). After 24 h in SM, the attached biomass remained stable up to 96 h whereas in LB the number of bacteria continuously decreased to 5.5 ± 3.3 10^8^ CFU (2.6 ± 1.6 10^5^ CFU/cm^2^) (Figure 3B). Therefore, the colonization of GW was fast and massive (above 10^7^ CFU/g GW at 20 min) and the colonization patterns were related to the initial bacterial concentration (Figure 3A,B). As in the total viable population, sessile bacteria survived over a longer time of incubation compared to planktonic counterparts, except in LB inoculated at 10^9^ CFU/mL (Figures 2 and 3).

We also examined fiber colonization by microscopy using an LB inoculum at 10^7^ CFU/mL (Figure 3C). After washing the GW, single and dispersed attached bacteria were visible after 1 h of incubation. At 3 h the density of the adherent cells had increased and at 6 h bacterial microcolonies were present on the fibers. These observations confirmed the previous result based on CFU follow-up (Figure 3A, LB medium).

### Adhesion was enhanced in adsorption mode compared to immersion mode

**Figure 4:**
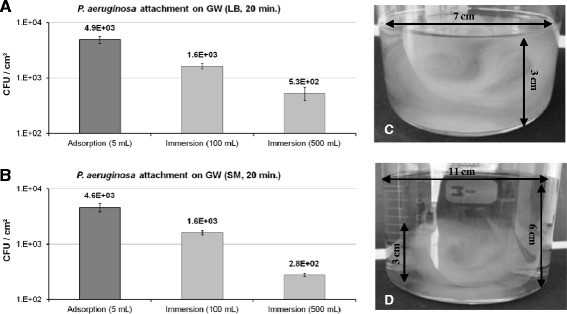
**Adsorption mode enhanced bacteria attachment compared to immersion mode.** We compared the number of CFU/cm^2^ on GW after 20 minutes of incubation at 37°C in LB **(A)** or SM **(B)** when GW was used in adsorption mode or immersion mode. In any case, a piece of 1 g GW was put in contact with ≈ 5.10^7^ CFU contained in 5 mL (adsorption); 100 mL or 500 mL (immersion) of medium. Pictures **C** and **D** illustrate the spreading of 1 g GW immersed in 100 mL and in 500 mL of LB, respectively.

The first step in the colonization of a surface is the physical contact between the bacteria and the substratum. In our adsorption system, the bacteria are in close contact with GW so the probability of cells being in contact with the surface should be greatly increased. For this reason, the attachment of *P. aeruginosa* on GW should be greater in adsorption mode than in immersion mode, where the bacteria are cultivated in the presence of GW immersed in a large volume of medium. To compare the colonization of the GW surface by *P. aeruginosa* in adsorption and immersion modes, we placed 1 g of GW in contact with the same number of bacteria (≈5 × 10^7^ CFU) diluted in 5 mL (adsorption mode) and in 100 mL or 500 mL (immersion mode) of LB or MS medium (Figure 4). After 20 min of incubation, GW was washed by using our flow-through process and the number of sessile bacteria was determined. To compare both modes, surface colonization was expressed as the number of sessile CFU/cm^2^ of surface covered by the medium. *P. aeruginosa* adhesion on GW was greater in the adsorption mode than in immersion mode (Figure 4A,B). The number of sessile bacteria / cm^2^ in the adsorption mode was 3-fold greater compared to immersion in 100 mL of LB or SM. When compared to immersion in 500 mL, it was 10- or 20-fold higher in LB or SM, respectively. *A priori*, greater differences were expected between the two modes. Our results might be due to the fact that GW expands readily in large volumes of medium (Figure 4C,D). For instance, 1 g of GW immersed in 100 mL of LB occupied almost the entire volume, which might have allowed the bacteria to easily encounter the substratum, especially for a highly motile bacterial strain like *P. aeruginosa*.

### Glass wool colonization by Pseudomonas aeruginosa involved protein synthesis

**Figure 5:**
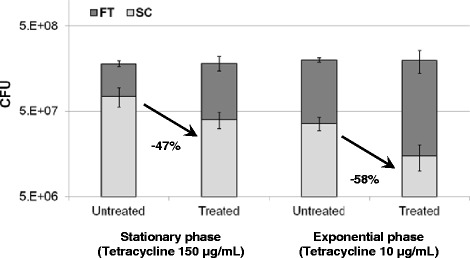
**Tetracycline effect on PAO1 GW colonization.** LB cultures were inoculated at 10^8^ CFU/mL. The cells were treated for 1 h with tetracycline at bacteriostatic concentration. Non-attached bacteria (FT, dark grey) and sessile bacteria (SC, grey) CFU were quantified after 20 min of incubation (see Materials and methods). Each point is the mean ± SD of biological triplicates.

Several publications have described differences in protein content between sessile and planktonic bacteria (summarized in [22]). Moreover, O’Toole and Kolter [21] described that protein synthesis is required for the initial steps of colonization in *P. fluorescens*. Using a similar protocol, we investigated the effect of tetracycline treatment on the initial colonization of GW by *P. aeruginosa*. For this experiment, tetracycline had to be used at a dose sufficient to inhibit protein synthesis but without any bactericidal effect. We determined that the tetracycline minimal inhibitory concentration of the PAO1 strain used was 10 μg/mL (data not shown). The bacteriostatic effect of tetracycline was verified on bacteria in exponential and stationary phases by treating a bacterial suspension at ≈ 10^8^ CFU/mL and counting CFU after incubation at 37°C. Although tetracycline at 150 μg/mL still had a bacteriostatic effect on stationary bacteria, a bactericidal effect was observed at 20 μg/mL on exponentially growing bacteria (65% reduction of viable cells after 20 min, data not shown). Therefore, experiments were performed with tetracycline at 150 μg/mL for stationary bacteria and 10 μg/mL for exponential bacteria. Tetracycline treatment of exponentially growing bacteria reduced the sessile population on GW by 58 ± 8% after 20 min of incubation, whereas the decrease was 47 ± 7% for stationary bacteria (Figure 5). This decrease was not related to the loss of PAO1 viability as the total number of viable bacteria remained the same after treatment with tetracycline. Protein synthesis was therefore necessary for initial attachment, a finding confirmed by using microplates and a crystal violet assay [see Additional file 5]. Once again, tetracycline treatment induced a 64 ± 3% reduction of the biofilm formed in wells after 20 min, whatever the physiological state of *P. aeruginosa*. In addition, the stationary bacteria were two-fold more adherent than their counterparts in the exponential phase (Figure 5; see Additional file 5).

### Adsorption mode allowed performing proteomic analysis of the first steps of colonization

**Figure 6:**
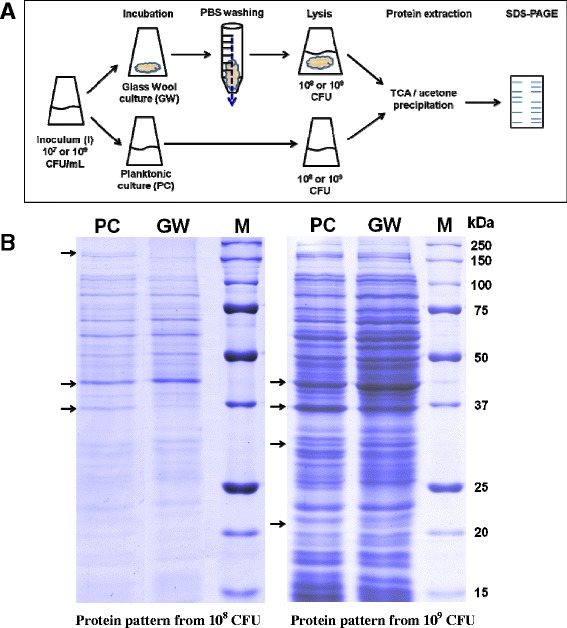
**Gel electrophoresis of proteins extracted from planktonic and sessile PAO1 bacteria. (A)** Bacterial suspensions were prepared at 10^7^ or 10^9^ CFU/mL from an overnight culture and cultivated for 3 h or 1 h at 37°C respectively in planktonic or adsorption mode. Proteins were directly extracted from 10^8^ or 10^9^ PAO1 planktonic cells (PC) and sessile bacteria attached to glass wool (GW). **(B)** Proteins were separated on SDS-PAGE (12%) and stained with a colloidal blue stain. Arrows indicate changes in protein pattern between PC and GW bacterial populations. M: Protein markers.

Since protein synthesis was involved in *P. aeruginosa* adhesion and because our system allowed the adhesion of a large amount of bacteria on GW within a very short time, we attempted to assess the protein content of sessile cells shortly after attachment. To do so, 1 g of GW was inoculated with 5 mL of LB at 10^7^ CFU/mL. After 3 h of incubation, 10^8^ sessile cells were directly lysed on GW. The same experiment was performed with LB at 10^9^ CFU/mL and 10^9^ sessile cells were lysed after 1 h of incubation. Similarly, bacteria (10^8^ or 10^9^ CFU) from planktonic cultures (PC) were directly lysed in the same buffer (Figure 6A). Then proteins were precipitated, separated by SDS-PAGE and stained with colloidal Coomassie blue (Figure 6B). Protein patterns were observed in all extracts, even at 10^8^ CFU, with proteins ranging from 15 to 250 kDa. GW and PC pattern intensities were comparable, indicating that most of the proteins were recovered in GW samples. Consistently, no residual proteins were extracted from GW fibers after a second treatment with hot 1% SDS (data not shown). Despite the low resolution of 1D-electrophoresis, we observed slight differences between GW and PC samples (Figure 6B). Thus, our system based on adsorption mode can be used to explore proteomic changes within the early stages of colonization without handling the sessile cells prior to lysis.

## Discussion

The development and validation of practical, reproducible and representative laboratory growth systems for the study of biofilms is a challenge. A panoply of *in vitro* systems have been developed, which range in complexity from a bacterial colony growing directly on agar plates to sophisticated continuous culture fermentation systems [23]. Each system has its own advantages, but the results of all these study systems are mixed. Biofilm physiology is dynamic [24] so analysis may be complicated by the action of several variables: nature of the substratum [25,26], the strain used and its degree of domestication [27], the carbon source [28], etc. For instance, a study has shown that surface hydrophobicity and charge have an effect on the initial colonization and attachment of cells onto the surface [29]. Biofilm systems that allow the growth rate to be controlled more accurately and heterogeneity to be minimized are arguably more suited to studies aimed at characterizing cell attachment and biofilm development. Therefore, biofilm formation systems can be considered in terms of the degree of control they provide over various aspects of physiology and the ease with which they can be established, maintained and replicated.

So far in our laboratory, *P. aeruginosa* biofilms have been grown on GW as substratum. GW was chosen as it affords a large surface-to-volume ratio for bacterial colonization [16]. Moreover, GW pieces are small and easy to handle. Conventionally, the surface is immersed in the culture medium and the sessile cells are collected, after washing and sonication [16,17,26]. Here we describe an improvement of our biofilm system. Instead of using the immersion mode, we tested the adsorption mode on GW. This makes it possible to work with a very small inoculum volume adsorbed and distributed over a large area and using a small piece of material. The fact that the culture volume was small did not impair bacterial growth. Furthermore, this configuration brought the bacteria near the support and enhanced their attachment to the surface. Comparison of the number of attached cells in immersion and adsorption modes showed the advantage of the latter system in obtaining more sessile biomass of *P. aeruginosa*. In adsorption mode, cell attachment was very efficient at short incubation times with more than 10^7^ sessile CFU within 20 min of incubation. The sessile bacteria population can be considered as corresponding to cells adhered to the surface, as our washing protocol (flow-through process) eliminated planktonic cells and cells loosely adhered to the substratum. In addition, analysis of the adherent cell population is not skewed by sessile cells attached to the recipient walls, as in the immersion mode. Thus, in a very short time, we obtained a large and presumably homogeneous population of sessile bacteria that had just adhered onto the GW. This new approach should reduce heterogeneity with respect to sessile biomass especially during the attachment period, and could pave the way for molecular analysis of the early events of biofilm formation.

During this study, we noticed that the initial colonization pattern changed reproducibly with the characteristics of the bacterial inoculum. Clearly the physiological state of *P. aeruginosa* cells had an impact on the degree of adhesion. Stationary cells were attached two-fold more than exponentially growing *P. aeruginosa*. Likewise the inoculum concentration affected the profile of colonization, irrespective of the culture medium. Whereas colonization regularly increased from the beginning with the concentrated inoculum (10^9^ CFU/mL), a kind of plateau occurred at the start of incubation (1 h – 3 h according to the medium used) with the more diluted inoculum (10^7^ CFU/mL). Nevertheless, these two distinct responses did not at first prevent the same maximal amount of biomass occurring in all experimental conditions. It was obvious that this plateau was not due to a limited surface adhesion capacity in that a larger number of bacteria were able to attach to GW when a higher bacterial concentration was used. In 2000, Rice and coauthors described a surface-associated lag time for *P. aeruginosa* PAO1 [30]. They analyzed the change from reversibly to irreversibly adsorbed cells on glass coverslips and the subsequent bacterial growth using confocal scanning laser microscopy. They found that *P. aeruginosa* cells that initially colonized the surface, also referred to as primary biofilm cells, experienced a lag in their growth; then the progeny of the first adhered population grew at the same rate as planktonic cells. They proposed that the lag phase occurred upon initial attachment of truly planktonic cells and was indicative of a physiological change from a planktonic to a sessile environment. Thus, attached cells at the plateau phase could correspond to a highly homogeneous population useful for the investigation of initial colonization. However, there are other reports where microorganisms do not exhibit a lag in replication after initial attachment [31,32]. These data underline the critical need for understanding the nature of the inoculum and for controlling the physiological state of cells used to generate the initial biofilm. Indeed in our experiments using a higher concentration from the same PAO1 pre-culture was sufficient to eliminate the lag phase.

Our results further demonstrate that protein synthesis is required for the early phase of GW colonization, whatever the inoculum produced from exponential or stationary *P. aeruginosa* cells. In both conditions, tetracycline treatment prevented about half of the bacteria from adhering to GW after 20 min incubation. This result was reproduced with microplates, suggesting that protein synthesis is necessary for starting colonization, irrespective of the surface properties. The involvement of protein synthesis has already been described in *P. fluorescens* [21]. The authors showed that the initial interaction with the abiotic surface required new protein synthesis but not for the subsequent step, *i.e.* the short-term maintenance of the attached cells. Our results and those of O’Toole and Kolter [21] suggest that the initial colonization phase in *Pseudomonas* is a regulated process involving the synthesis of new proteins. This observation seems consistent with the processes that may involve hydrodynamic and physical-chemical interactions in the deposition of cells onto the surface [33].

Understanding the transition from the deposition stage, during which there is potential for bacterial removal, to the development of irreversible interactions with the surface is a way to develop strategies for preventing biofilm formation. Our biofilm system may make it possible to explore the early stages and to identify the molecular components by which bacteria deposit and, shortly after, attach irreversibly to surfaces. To demonstrate the potential of our biofilm system for analyzing bacterial adhesion, we tried to visualize the protein content of a small amount of sessile cells after 1 h or 3 h incubation. A very critical issue in sample preparation is the need to rapidly and efficiently quench all biological and enzymatic activities in order to capture an accurate “snap-shot” of the proteome. Rapid cell lysis should avoid changes in gene expression that result from the process of harvesting the cells and might perturb their state. Our system makes it possible to directly lyse sessile cells on their support and immediately freeze their protein content. The initial results showed that proteins extracted from a small amount of sessile bacteria (10^8^ CFU) were clearly reachable on 1-D polyacrylamide gel. A comparison with the protein profile obtained from the same amount of planktonic bacteria reproducibly revealed some differences. These differences were consistent with the involvement of protein synthesis in the initial colonization of GW. These preliminary results show that the *P. aeruginosa* biofilm proteome can be studied soon after inoculation of GW either by SDS-PAGE analysis or another tool such as mass spectrometry. Indeed, mass spectrometry is more sensitive and requires a smaller amount of proteins compared with SDS-PAGE. It also covers a wider diversity of the proteome. Beyond the above-mentioned characteristics of our biofilm system, the adsorption mode on GW is easy to perform, reliable and adjustable. The physiological response of the organism tested by manipulation of a single variable can thus be easily monitored. The reduction of incubation volume can also be seen as an advantage when working with expensive media cultures. Furthermore, this tool can be miniaturized to monitor biofilm formation in a wide variety of experimental conditions. Finally, this new approach can be extended to other bacterial organisms.

## Conclusions

So far, the literature did not report any molecular studies describing the very first steps of biofilm formation, in particular bacterial attachment. Our experimental approach based on adsorption of the medium onto GW fibers offers the opportunity to perform molecular and proteomic analysis of the early stages of colonization, in particular within the first hour of incubation. We showed that the colonization of the GW surface is very fast, irrespective of the medium, and depends on protein synthesis. Then our growth system should permit identification of proteins involved in cell attachment and decipher the very early steps in biofilm formation.

## Additional files

Additional file 1:
**Characteristics of 1 g piece of glass wool (GW).** (A) Sizes of 1 g GW piece (Ø and h). (B) Determination of the GW fiber diameter by optical microscopy (10 μm; n = 10). (C) The GW density (1154.7 ± 57.6 kg/m3) and the fiber diameter allowed the schematic representation of 1 g of GW as a cylinder of 11 × 103 m length offering a surface of 3464 cm^2^. Additional file 2:
**Surface covered by the medium adsorbed on a 1 g piece of glass wool.** The maximum volume that could be loaded on 1 g of GW was determined to be 10 mL. Based on this, we examined by methylene blue staining (see Methods section) the % of surface covered as a function of the volume adsorbed on GW. The photos A, C, E present top views and B, D, F vertical section views. A ratio of 10 or 7.5 mL/g GW allowed covering 100% of the surface (not shown); 5 mL/g GW 61.0 ± 1.4% (A, B); 2.5 mL / g GW 30.0 ± 2.0% (C, D) and 1 mL/g GW 13.7 ± 0.8% (E, F). Data were obtained from 6 independent experiments. Additional file 3:
**Determination of the thickness of medium adsorbed on a 1 g piece of glass wool.** Using a hollow cylinder model (A), we calculated the theoretical thickness of a liquid adsorbed on GW without taking into account the % of covered surface (B; blue line) and taking into account the experimentally determined % of covered surface (B; red line). We determined the existence of a minimal theoretical thickness, relatively constant and calculated to be ≈ 13 μm (B). Additional file 4:
**Washing process of glass wool allowing separation of strongly attached bacteria from planktonic and weakly attached bacteria.** Washing was performed by 100 mL of PBS going by gravity through the GW set in a syringe. Bacteria were recovered and quantified by plating on agar plates. The calibrated inoculum consisted of LB or SM at 107, 108 or 109 CFU/mL was also quantified for comparison. In every case, more than 98% of the inoculated bacteria were harvested in the PBS when GW was treated immediately after inoculation. Additional file 5:
**Tetracycline effect on PAO1 cells attachment in GW and microplate systems.** The LB cultures were inoculated at 108 CFU/mL. The cells were treated for 1 h with tetracycline at bacteriostatic concentration before quantifying the sessile population after 20 min incubation (see Methods section). Effect of tetracycline was assayed on GW in adsorption mode (A, data from Figure 5) and in 96-wells plates (B). Each point is the mean ± SD of biological triplicates.
