# Supplementary material for: Exploring early steps in biofilm formation: set-up of an experimental system for molecular studies
Source: BMC Microbiol. 2014 Sep 30;14:253. doi: 10.1186/s12866-014-0253-z (PMC4189659; doi:10.1186/s12866-014-0253-z)
Supplement: Additional file 1: — Characteristics of 1 g piece of glass wool (GW). (A) Sizes of 1 g GW piece (Ø and h). (B) Determination of the GW fiber diameter by optical microscopy (10 μm; n = 10). (C) The GW density (1154.7 ± 57.6 kg/m3) and the fiber diameter allowed the schematic representation of 1 g of GW as a cylinder of 11 × 103 m length offering a surface of 3464 cm2. [file 12866_2014_253_MOESM1_ESM.pdf]

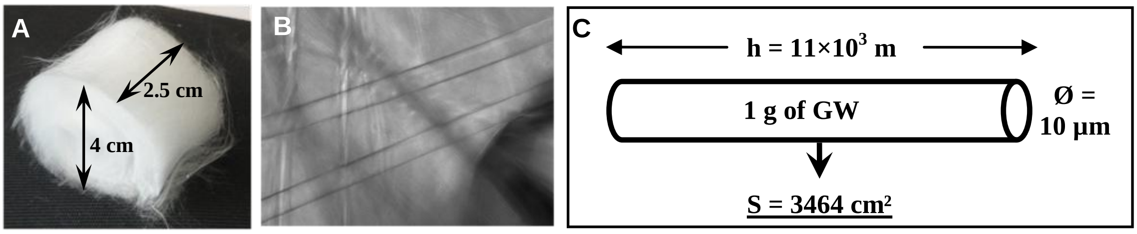

Additional file 1: Characteristics of 1g piece of glass wool (GW). (**A**) Sizes of 1g GW piece (Ø and h). (**B**) Determination of the GW fiber diameter by optical microscopy (10 µm; n = 10). (**C**) The GW density (1154.7 ± 57.6 kg/m^3) and the fiber diameter allowed the schematic representation of 1g of GW as a cylinder of 11×10^3 m length offering a surface of 3464 cm².
