# Supplementary material for: Exploring early steps in biofilm formation: set-up of an experimental system for molecular studies
Source: BMC Microbiol. 2014 Sep 30;14:253. doi: 10.1186/s12866-014-0253-z (PMC4189659; doi:10.1186/s12866-014-0253-z)
Supplement: Additional file 2: — Surface covered by the medium adsorbed on a 1 g piece of glass wool. The maximum volume that could be loaded on 1 g of GW was determined to be 10 mL. Based on this, we examined by methylene blue staining (see Methods section) the % of surface covered as a function of the volume adsorbed on GW. The photos A, C, E present top views and B, D, F vertical section views. A ratio of 10 or 7.5 mL/g GW allowed covering 100% of the surface (not shown); 5 mL/g GW 61.0 ± 1.4% (A, B); 2.5 mL / g GW 30.0 ± 2.0% (C, D) and 1 mL/g GW 13.7 ± 0.8% (E, F). Data were obtained from 6 independent experiments. [file 12866_2014_253_MOESM2_ESM.pdf]

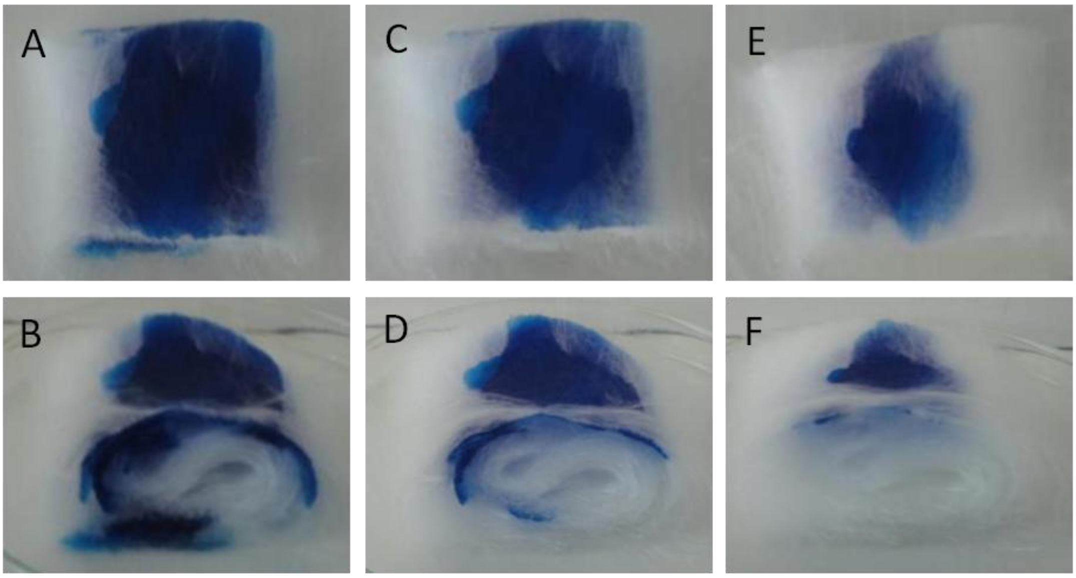

Additional file 2: Surface covered by the medium adsorbed on a 1g piece of glass wool. The maximum volume that could be loaded on 1g of GW was determined to be 10 mL. Based on this, we examined by methylene blue staining (see methods section) the % of surface covered as a function of the volume adsorbed on GW. The photos A, C, E present top views and B, D, F vertical section views. A ratio of 10 or 7.5 mL / g GW allowed covering 100% of the surface (not shown); 5 mL / g GW 61.0 ± 1.4% (**A, B**); 2.5 mL / g GW 30.0 ± 2.0% (**C, D**) and 1 mL / g GW 13.7 ± 0.8 % (**E, F**). Data were obtained from 6 independent experiments.
