# Supplementary material for: Exploring early steps in biofilm formation: set-up of an experimental system for molecular studies
Source: BMC Microbiol. 2014 Sep 30;14:253. doi: 10.1186/s12866-014-0253-z (PMC4189659; doi:10.1186/s12866-014-0253-z)
Supplement: Additional file 3: — Determination of the thickness of medium adsorbed on a 1 g piece of glass wool. Using a hollow cylinder model (A), we calculated the theoretical thickness of a liquid adsorbed on GW without taking into account the % of covered surface (B; blue line) and taking into account the experimentally determined % of covered surface (B; red line). We determined the existence of a minimal theoretical thickness, relatively constant and calculated to be ≈ 13 μm (B). [file 12866_2014_253_MOESM3_ESM.pdf]

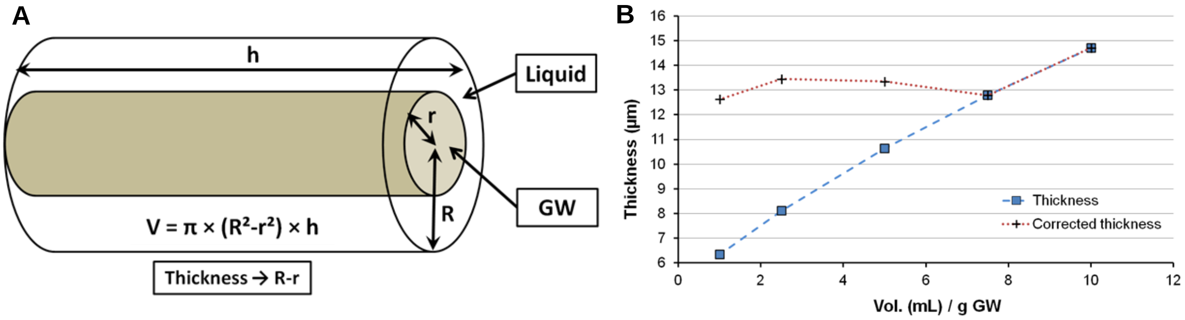

Additional file 3: Determination of the thickness of medium adsorbed on a 1g piece of glass wool. Using a hollow cylinder model (**A**), we calculated the theoretical thickness of a liquid adsorbed on GW without taking into account the % of covered surface (**B**; blue line) and taking into account the experimentally determined % of covered surface (**B**; red line). We determined the existence of a minimal theoretical thickness, relatively constant and calculated to be ≈ 13 µm (**B**).
