# Supplementary material for: Exploring early steps in biofilm formation: set-up of an experimental system for molecular studies
Source: BMC Microbiol. 2014 Sep 30;14:253. doi: 10.1186/s12866-014-0253-z (PMC4189659; doi:10.1186/s12866-014-0253-z)
Supplement: Additional file 4: — Washing process of glass wool allowing separation of strongly attached bacteria from planktonic and weakly attached bacteria. Washing was performed by 100 mL of PBS going by gravity through the GW set in a syringe. Bacteria were recovered and quantified by plating on agar plates. The calibrated inoculum consisted of LB or SM at 107, 108 or 109 CFU/mL was also quantified for comparison. In every case, more than 98% of the inoculated bacteria were harvested in the PBS when GW was treated immediately after inoculation. [file 12866_2014_253_MOESM4_ESM.pdf]

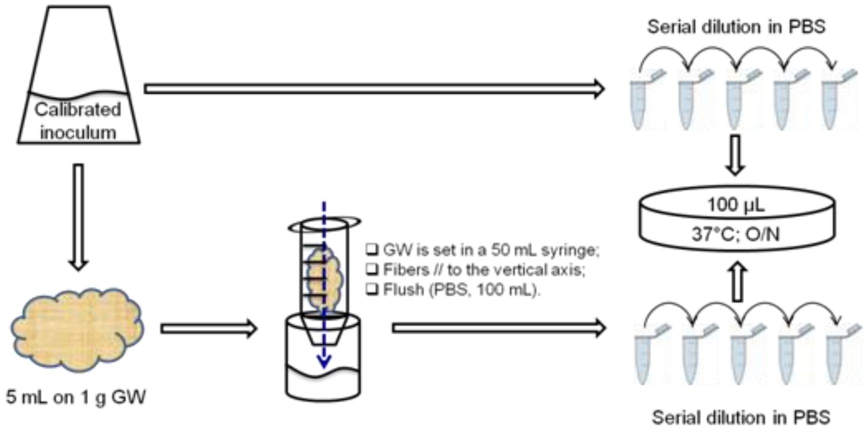

Additional file 4: Washing process of glass wool allowing separation of strongly attached bacteria from planktonic and weakly attached bacteria. Washing was performed by 100 mL of PBS going by gravity through the GW set in a syringe. Bacteria were recovered and quantified by plating on agar plates. The calibrated inoculum consisted of LB or SM at 10^7, 10^8 or 10^9 CFU/mL was also quantified for comparison. In every case, more than 98% of the inoculated bacteria were harvested in the PBS when GW was treated immediately after inoculation.
