# Supplementary material for: Exploring early steps in biofilm formation: set-up of an experimental system for molecular studies
Source: BMC Microbiol. 2014 Sep 30;14:253. doi: 10.1186/s12866-014-0253-z (PMC4189659; doi:10.1186/s12866-014-0253-z)
Supplement: Additional file 5: — Tetracycline effect on PAO1 cells attachment in GW and microplate systems. The LB cultures were inoculated at 108 CFU/mL. The cells were treated for 1 h with tetracycline at bacteriostatic concentration before quantifying the sessile population after 20 min incubation (see Methods section). Effect of tetracycline was assayed on GW in adsorption mode (A, data from Figure 5) and in 96-wells plates (B). Each point is the mean ± SD of biological triplicates. [file 12866_2014_253_MOESM5_ESM.pdf]

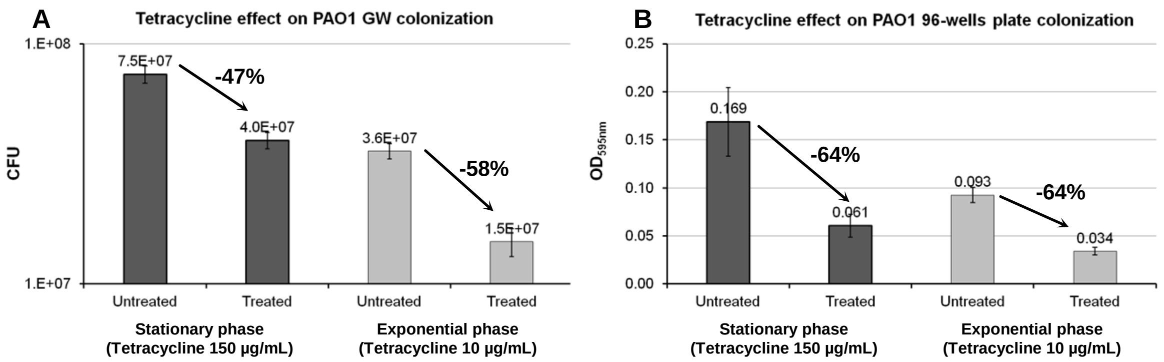

Additional file 5: Tetracycline effect on PAO1 cells attachment in GW and microplate systems. The LB cultures were inoculated at 10^8 CFU/mL. The cells were treated for 1h with tetracycline at bacteriostatic concentration before quantifying the sessile population after 20 min incubation (see methods section). Effect of tetracycline was assayed on GW in adsorption mode (**A,** data from Figure 5) and in 96-wells plates (**B**). Each point is the mean ± SD of biological triplicates.
